# Single-nucleotide polymorphisms and activities of indoleamine 2,3-dioxygenase isoforms, IDO1 and IDO2, in tuberculosis patients

**DOI:** 10.1186/s41065-022-00219-y

**Published:** 2022-01-19

**Authors:** Tingming Cao, Guangming Dai, Hongqian Chu, Chengcheng Kong, Huijuan Duan, Na Tian, Zhaogang Sun

**Affiliations:** 1 Beijing Key Laboratory of Drug-Resistant Tuberculosis Research, Beijing Tuberculosis and Thoracic Tumor Research Institute, 9 Beiguan Street, Tongzhou District, Beijing, 101149 China; 2 Translational Medicine Center, Beijing Chest Hospital, Capital Medical University, Beijing, 101149 China

**Keywords:** Indoleamine 2,3-dioxygenase, Tryptophan, Kynurenine, Single-nucleotide polymorphism

## Abstract

**Purpose:**

To explore the role and effects of the single-nucleotide polymorphisms (SNPs) of the two functionally related indoleamine 2,3-dioxygenase (IDO) isoforms on IDO activity in the Chinese Han ethnic population.

**Methods:**

A total of 151 consecutive patients of Chinese Han ethnicity (99 men and 52 women; average age 51.92 ± 18.26 years) with pulmonary TB admitted to Beijing Chest Hospital between July 2016 and February 2017 were enrolled in the study. The serum levels of tryptophan (Trp) and its metabolites, *IDO1* and *IDO2* mRNA levels, and the relationship of *IDO1* and *IDO2* SNPs with the serum Kyn/Trp ratio in TB patients and healthy controls were examined by LC/ESI–MS/MS analysis. Genomic DNA was isolated from whole blood, and the PCR products were sequenced and analyzed.

**Results:**

In Chinese Han participants, only *IDO2* had SNPs R248W and Y359X that affected IDO activity, as determined by the serum Kyn/Trp ratio. *IDO1* and *IDO2* mRNA levels were inversely related in TB patients and healthy controls.

**Conclusions:**

*IDO2* SNPs and the opposite expression pattern of *IDO1* and *IDO2* affected IDO activity in Chinese Han TB patients.

## Introduction

Indoleamine 2,3-dioxygenase (IDO) is a widely expressed inducible enzyme. IDO comprises two alpha helical domains with a heme group between them. It contributes to the tryptophan (Trp) catabolic pathway and converts Trp to N-formyl kynurenine (Kyn) [1]. Trp depletion by IDO can have detrimental consequences on cell function and survival, especially in immune response and neurotransmission [2, 3]. The two IDO isoforms, IDO1 and IDO2, are functionally related and have 43% sequence identity. They are diverse in their expression [4, 5], enzyme activities [6, 7], regulatory impacts [6, 8], and biochemical effects [9, 10].

In addition, IDO activity plays a crucial role in immune tolerance to pathogens. In a macaque infection model of tuberculosis (TB), the suppression of IDO activity reduced bacterial burden, pathology, and clinical signs, leading to increased host survival [11]. The IDO–Kyn pathway is involved in TB by inducing *IDO1* expression and activating Trp metabolism [12]. IDO activity has been assessed in serum [13] and pleural fluid [14], and, with a significant increase in the serum Kyn/Trp ratio, it can be used as a biomarker for TB patients infected with human immunodeficiency virus [15] and for patients with multidrug-resistant TB [16].

Single-nucleotide polymorphisms (SNPs) in the promoters or coding regions of *IDO1* and *IDO2* may affect their enzyme activities. SNPs R248W and Y359X in the coding sequence of *IDO2* can attenuate catalytic activity [17] and are potential diagnostic markers for multiple myeloma [18], although they are not associated with *Aspergillus fumigatus* infection in patients with cystic fibrosis [19]. SNPs in the *IDO1* gene c. -1493G > C - *IDO1* (rs10089084) and c. -1849C > A - *IDO1* (rs3824259) have been linked to depression [20]. In addition, the rs3808606 T/T genotype has been correlated with reduced susceptibility to recurrent vulvovaginal candidiasis [21]. However, the SNPs in IDO1 and IDO2 were reported in different regions and countries, including Austrailia [22], Brazil [23], india [21], Italy [19], Japan [18], Poland [20] and United States of America [24].

China is a multi-ethnic country with about 55 ethnic groups, and the Han ethnic group is the largest. Therefore, *IDO1* and *IDO2* SNPs in TB patients of Chinese Han ethnicity were investigated in this study. In addition, serum Trp and its metabolites were analyzed to explore the individual roles of *IDO1* and *IDO2* SNPs in affecting the serum Kyn/Trp ratio. Furthermore, *IDO1* and *IDO2* mRNA levels were determined by quantitative real-time PCR (qRT-PCR) in healthy controls, TB patients, and infected mice. Finally, the study investigated the relationship of *IDO1* and *IDO2* SNPs with the serum Kyn/Trp ratio.

## Materials and methods

### Participants

A total of 151 consecutive patients of Chinese Han ethnicity (99 men and 52 women; average age 51.92 ± 18.26 years) with pulmonary TB admitted to Beijing Chest Hospital between July 2016 and February 2017 were enrolled in the study. Pulmonary TB was diagnosed by the rapid culture method using Bactec MGIT 960 (BD, Sparks, MD, USA). The study also included 132 participants of Chinese Han ethnicity (83 men and 49 women; average age 47.21 ± 13.20 years) recruited in the hospital as a healthy control group. This study was approved by the Ethics Committee of Beijing Chest Hospital (approval number 2016–05), and written informed consent was obtained in accordance with the hospital’s guidelines.

### Serum preparation

Blood samples from 64 TB patients were drawn at the time of admission before the start of TB treatment. During routine laboratory examinations, patient serum samples were collected and frozen at − 80 °C until analysis. Blood and serum samples were also collected from 60 healthy participants.

### Measurement of serum Trp metabolites

Before analyzing serum Trp and its metabolites in the IDO–Kyn pathway, the standard curves of pure Trp and Kyn (Sigma–Aldrich, St. Louis, MI, USA) were obtained by liquid chromatography/electrospray ionization–tandem mass spectrometry (LC/ESI–MS/MS) according to the method of Suzuki et al. [13, 14]. Briefly, frozen serum samples were thawed at room temperature for deproteinization with acetonitrile (50 μL of serum with 50 μL of deionized water and 100 μL of acetonitrile) on ice for 5 min. The samples were centrifuged (15,000×*g*; 10 min; 4 °C) and filtered with a 0.22-mm filter. Further, 100 mL of supernatant was used for auto-filling. LC/ESI–MS/MS analysis was performed using an Agilent 6410A triple quadrupole mass spectrometer equipped with an ESI interface coupled to an Agilent 1260 UHPLC system (Agilent Technologies, CA, USA). Chromatographic separation for the analysis was performed in the isocratic mode on a Zorbax SB-Aq analytical column (2.1 × 100 mm^2^; particle size, 3.5 μm). The mobile phase of acetonitrile:deionized water (0.1% formic acid) [30:70 (*v*/*v*)] was passed at a flow rate of 0.3 mL/min. Detection was performed using electrospray MS/MS in the multiple reaction monitoring mode (ionization voltage, 4000 V; temperature of MS1 and MS2, 100 °C; atomization pressure, 35 psi; drying gas flow rate, 10 L/min). IDO activity was determined by the serum Kyn/Trp ratio.

LC/ESI–MS/MS analysis data were obtained using the Agilent MassHunter Workstation software and were further analyzed using the Agilent MassHunter Quantitative Analysis software. The actual quantity (μmol/L) of Trp and Kyn in serum was calculated based on the quantitative standard curve obtained by chromatographic analysis.

### DNA preparation and SNP analysis

Genomic DNA was isolated from whole blood using a commercially available DNA extraction kit (Bioteke, Wuxi, China), and the concentration was determined by measuring the absorbance at 260 and 280 nm. The isolated DNA sample was stored at − 20 °C until analysis. The primer sequences are listed in Table 1. The PCR products were sequenced by Sangon Biotech (Shanghai, China), and the results were analyzed using the MegAlign software (DNASTAR Inc., Madison, WI, USA).

**Table 1:** SNP and qRT-PCR primers of *IDO1* and *IDO2*

Primer	Primer sequences (5′–3′)	PCR purpose
*IDO1*-Exon2&3	GAAGGCAAGGCATACTATCAG	SNPs
	GGAAAGTTAAATGTAAATTAGATG	
*IDO1*-Exon4	CAGGAGCAAGACTCCATCTC	SNPs
	GTAGTGGTAGACACAGCAGTC	
*IDO1*-Exon6	GATAGTAAGGCCTGCCACAC	SNPs
	GTTTAGGCTCCGAAGTGATTG	
*IDO1*-Exon7	CTGGACAACTGAGCGAGACTC	SNPs
	CTATTCTACACCTGGAACATTTG	
*IDO2*-R248W	GAACATTCTATCCCCCGTTGC	SNPs
	TTACCTGAGAGTGGATCCCTAGCA	
*IDO2*-Y359X	TCTTGTGCTCCCTCCAAAACA	SNPs
	TGGTTTGGCTTCCCATGCTT	
Human *IDO1*	GATGTCCGTAAGGTCTTGCCA	qRT-PCR
	TGCAGTCTCCATCACGAAATG	
Human *IDO2*	CTGATCACTGCTTAACGGCA	qRT-PCR
	TGCCACCAACTCAACACATT	
Human β-actin	TGGCACCCAGCACAATGAA	qRT-PCR
	CTAAGTCATAGTCCGCCTAGAAGC	
Mouse *IDO1*	ATCGC AGCTT CTCCTGCAAT	qRT-PCR
	TGTAT CGATG GTCAGACCTC	
Mouse *IDO2*	A TA CTG GCC TCT GGT CCT	qRT-PCR
	GGTGGCAGC GGA GAT AAT	
Mouse β-actin	AGCCATGTACGTAG CCATCC	qRT-PCR
	CTCTCAGCTGTGGTGGTGAA	

### Mouse infection experiments

For the mouse infection model, 16 female BALB/c mice (18–20 g) were purchased from the Laboratory Animal Institute of Chinese Academy of Medical Science (Beijing, China) and were housed according to the animal welfare regulations of Beijing Chest Hospital. The mice were infected with ~ 300 colony-forming units of *Mycobacterium tuberculosis* H37Rv (ATCC 27294) by the aerosol route (099C A4224 Inhalation Exposure System; Glas-Col, Terre Haute, IN, USA) in an animal biosafety level 3 facility. Blood from the retro-orbital plexus was collected at 1, 2, 3, and 4 weeks for total RNA isolation.

### RNA isolation and qRT-PCR

Total RNA was extracted and reverse transcribed into cDNA using an RNeasy Mini Kit and a Qiagen One-Step RT-PCR Kit (Qiagen, Shanghai, China) following the manufacturer’s protocols. qRT-PCR was performed using cDNA as a template and SYBR Green I as the fluorescent dye. Amplification efficiencies were validated and normalized against human and mouse β-actin. The qRT-PCR program was as follows: 95 °C for 3 min, followed by 40 cycles of denaturation at 95 °C for 30 s, and an annealing/extension step at 58 °C for 30 s. The primer sequences are listed in Table 1.

### Bioinformatics and statistical analyses

The three-dimensional (3D) protein structures of IDO1 and IDO2 were predicted using the SWISS-MODEL server, and the interaction values of binding free energy (ΔG) and dissociation constant (*K*_d_) were estimated using the PPA-Pred2 online software (Protein-Protein Affinity Predictor; https://www.iitm.ac.in/bioinfo/PPA_Pred/prediction.html). Genotypic and allelic frequencies of *IDO1* and *IDO2* in TB patients and healthy controls were compared using the *chi*-square test. The levels of Trp metabolites, Treg cells, and Th17 cells in TB patients and healthy controls were compared using the independent-samples *t-*test for continuous variables.

## Results

### *IDO1* and *IDO2* SNPs in Chinese Han participants

**Fig. 1:**
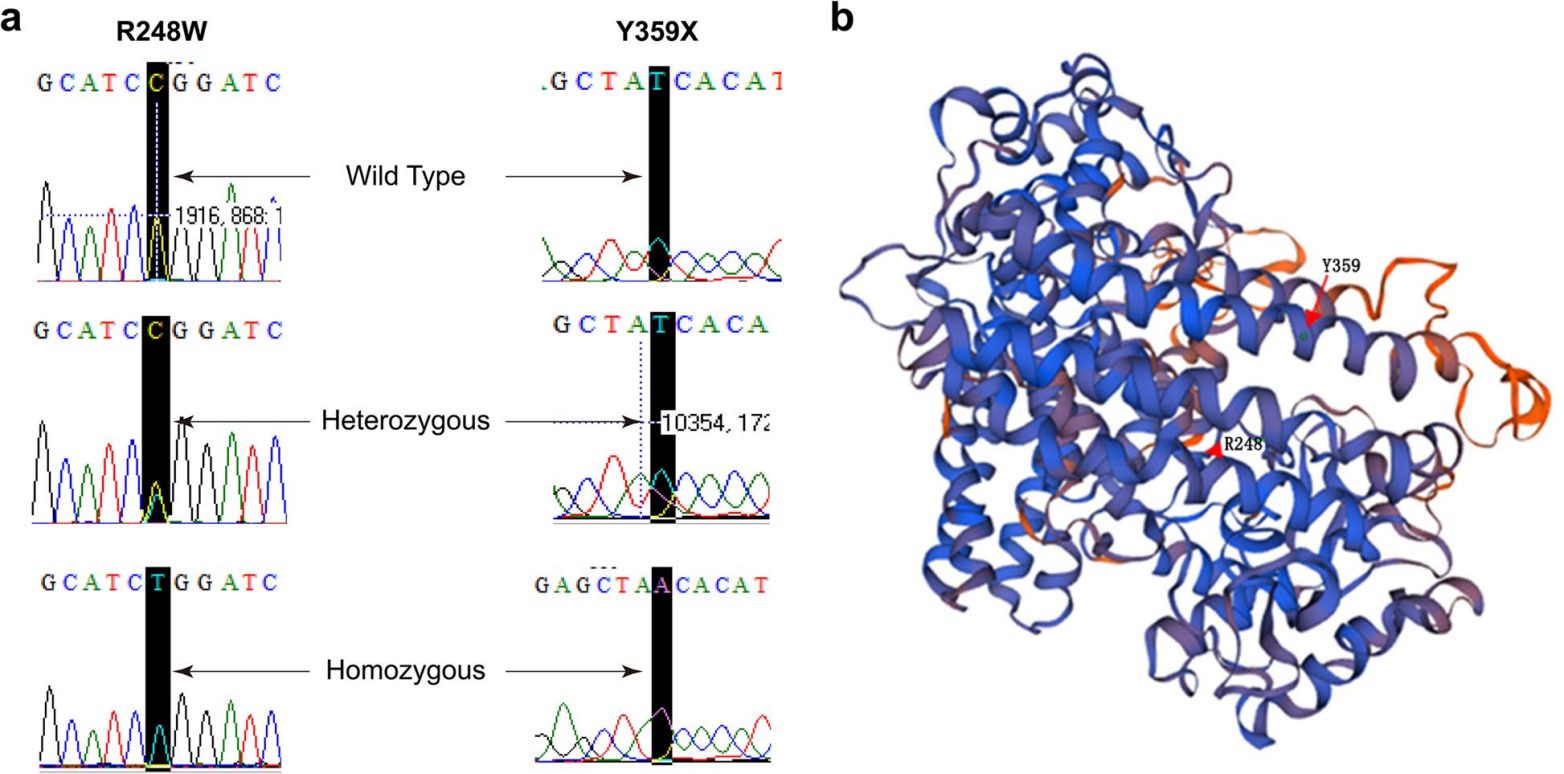
Sanger sequence electropherograms of R248W and Y359X and the position of R248W and Y359X in the predicted 3D structure. (a) Three *IDO2* genotypes were found in R248W and Y359X: wild type, heterocigoto type, and homocigoto type; (b) Mutations in the predicted 3D structure of IDO2 (red arrows). Structure prediction using the SWISS-MODEL server showed that IDO1 and IDO2 formed dimers. Interaction values of binding free energy (− 12.75 kcal/mol) and dissociation constant (4.50 e^− 10^ M) were estimated using the PPA-Pred2 online software (Protein-Protein Affinity Predictor; https://www.iitm.ac.in/bioinfo/PPA_Pred/prediction.html)

Previous studies reported polymorphisms I91L, A196T, F222S, Q272K, A277D, and S284P in IDO1, which consists of 403 amino acids [22–25]. In this study, no *IDO1* SNP was found in Chinese Han participants. For *IDO2*, SNPs R248W and Y359X were found in both TB patients and healthy controls as in previous reports [6, 26] **(**Fig. 1**;** Table 2), which was in agreement with Hardy–Weinberg equilibrium. The T allele frequency of R248W was 0.28 in TB patients and 0.31 in healthy controls. The A allele frequency of Y359X was 0.39 in TB patients and 0.35 in healthy controls. Both T allele frequency of R248W (*P* = 0.40) and A allele frequency of Y359X (*P* = 0.35) showed no significant difference between TB patients and healthy controls. No significant difference was found in the distribution of genotypes (*P*_*R248W*_ = 0.25, *P*_*Y359X*_ = 0.28) between TB patients and healthy controls.

**Table 2:** Genotypic and allelic frequencies of *IDO2*-R248W and *IDO2*-Y359X in TB patients and healthy controls

	R248W (*n*, %)				Y359X (*n*, %)			
	WT (CC)	Heterocigoto (CT)	Homocigoto (TT)	T allele frequency	WT (TT)	Heterocigoto (TA)	Homocigoto (AA)	A allele frequency
Healthy controls (*n* = 132)	66 (50.0)	51 (38.6)	15 (11.4)	0.31	60 (7.6)	51 (87.1)	21 (5.3)	0.35
Patients (*n* = 151)	77 (51.0)	65 (43.0)	9 (6.0)	0.28	56 (37.1)	72 (47.7)	23 (15.2)	0.39
Total (*n* = 283)	143 (12.0)	116 (81.3)	24 (6.7)	0.29	116 (41.0)	123 (43.5)	44 (15.5)	0.37
*P*-value	0.25			0.40	0.28			0.35

### *IDO1* and *IDO2* mRNA levels in TB patients and infected mice

**Fig. 2:**
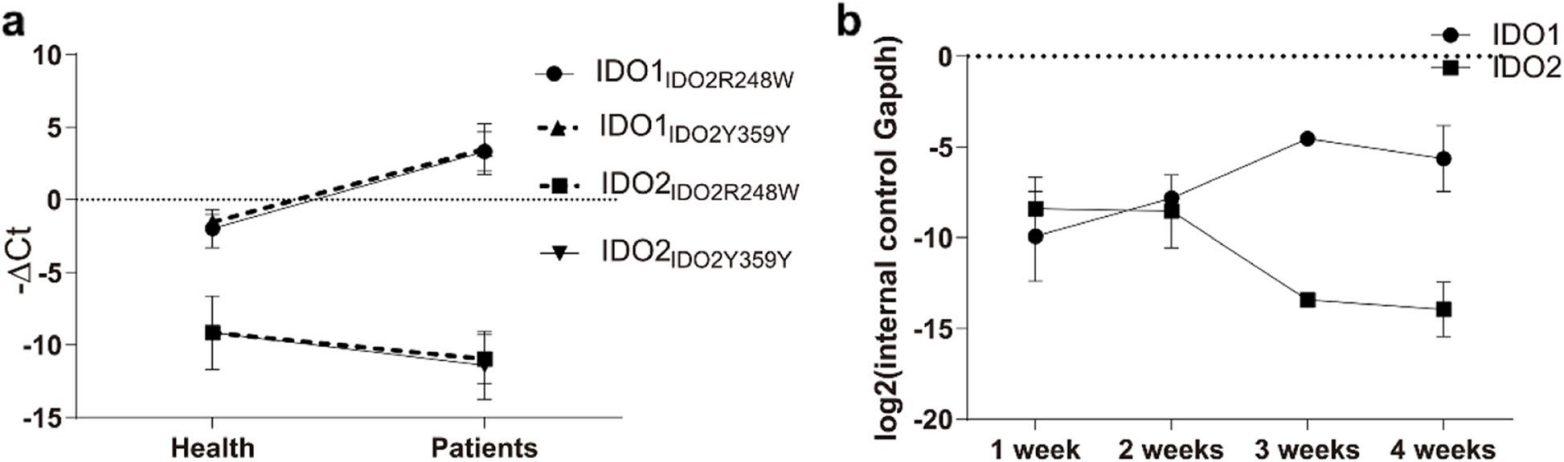
*IDO1* and *IDO2* mRNA levels in healthy controls, TB patients, and *M. tb* H37Rv-infected BALB/c mice. **a** *IDO1* and *IDO2* mRNA levels in TB patients and healthy controls grouped by *IDO2* genotypes; (**b**) *IDO1* and *IDO2* mRNA levels in *M. tb* H37Rv-infected BALB/c mice with four in each group. The mice were infected with 300 colony-forming units by the aerosol route

*IDO1* and *IDO2* mRNA levels were determined by qRT-PCR. *IDO1* expression in both *IDO2*-R248W and *IDO2*-Y359X groups was highly upregulated in TB patients than in healthy controls. *IDO2* expression in both *IDO2*-R248W and *IDO2*-Y359X groups was downregulated in TB patients (Fig. 2a). To confirm the differential expression of *IDO1* and *IDO2* in TB infection, their mRNA levels were assessed in *M. tuberculosis* H37Rv-infected BALB/c mice. *IDO1* mRNA levels in infected mice continued to increase for 3 weeks after infection, whereas *IDO2* mRNA levels showed an opposite trend (Fig. 2b).

Considering the different *IDO2* genotypes in *IDO1* and *IDO2* expression, *IDO1* mRNA levels in *IDO2*-R248W and *IDO2*-Y359X groups were not significantly different in both TB patients and healthy controls. In addition, no difference was found for *IDO2* expression in the *IDO2*-R248W and *IDO2*-Y359X groups in both TB patients and healthy controls.

### Serum levels of Trp and its metabolites and the Kyn/Trp ratio in different *IDO2* genotypes

**Fig. 3:**
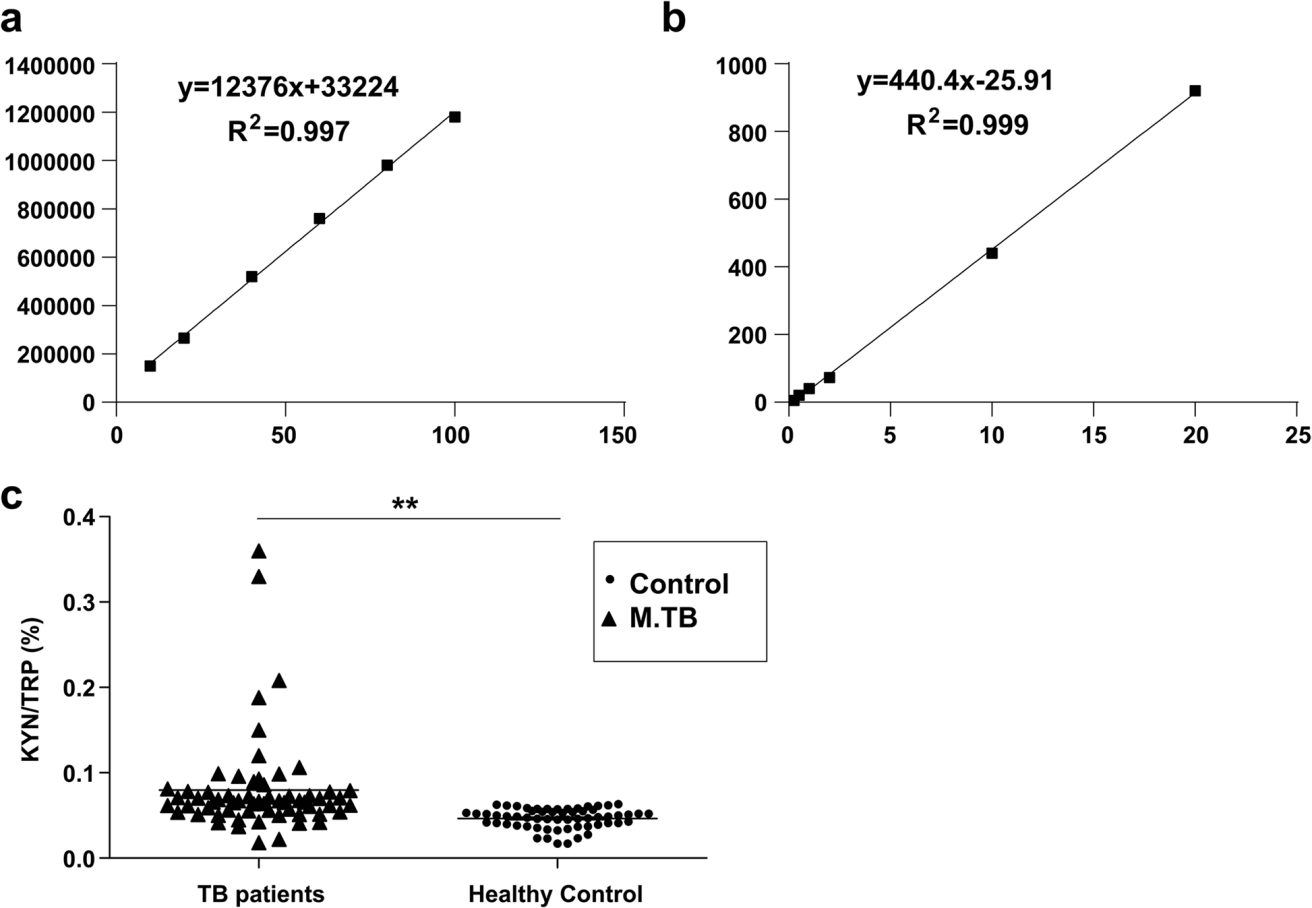
Quantitative standard curves by chromatographic analysis (LC/ESI–MS/MS). **A** Serum Trp; (**B**) Serum Kyn; (**C**) Serum Kyn/Trp ratio in TB patients and healthy controls. *P < 0.05

LC/ESI–MS/MS was used to obtain the standard curves and concentration equations for calculating Kyn, Trp, and Trp metabolites in TB patients and healthy controls (Fig. 3). Using these equations, the serum levels of Trp and its metabolites were calculated (Table 3). Overall, a negative linear correlation was observed between Trp and Kyn serum levels. IDO activity determined by the serum Kyn/Trp ratio was significantly higher in TB patients than in healthy controls [lower Trp concentration (*P* = 0.002) and higher Kyn concentration (*P* = 0.000); Table 3].

**Table 3:** Serum levels of Trp and its metabolites in healthy controls and TB patients (μmol/L)

Group	*n*	Kyn/Trp	Kyn	Trp	PA	QA	AA
Healthy controls	60	0.03 ± 0.01	1.70 ± 0.35	52.55 ± 10.45	0.07 ± 0.04	1.95 ± 0.88	0.69 ± 0.03
TB patients	64	0.06 ± 0.03^*^	2.42 ± 1.02^*^	48.18 ± 16.56^*^	0.08 ± 0.05	8.04 ± 10.33^*^	0.71 ± 0.08

^*^Significant differences between TB patients and healthy controls, *P <* 0.05

To assess the effects of *IDO2* SNPs on IDO activity, the serum Kyn/Trp ratios of wild type (WT) R248W(TT) versus Homocigoto mutation R248W(CC) and WT Y359X(AA) versus Homocigoto mutation Y359X(TT) were compared. Both SNPs R248W and Y359X significantly affected IDO activity (Table 4). The Homocigoto mutation R248W(CC) exhibited lower IDO activity (Kyn/Trp = 0.053 ± 0.031) than WT R248W(TT) (Kyn/Trp = 0.061 ± 0.029). The Homocigoto mutation Y359X(TT) exhibited higher IDO activity (0.052 ± 0.033) than the average (0.050 ± 0.028), and it affected IDO activity more than the Homocigoto mutation Y359X(AA) (*P* = 0.021). Patients with R248W(TT) showed the highest serum Kyn/Trp ratio (0.061 ± 0.029) compared to patients with the other three SNPs.

**Table 4:** Kyn and Trp serum levels in healthy controls and TB patients with R248W and Y359X genotypes (μmol/L)

Group	Healthy controls	Patients	R248W(TT) in patients	R248W(CC) in patients	Y359X(TT) in patients	Y359X(AA) in patients
*N*	60	64	10	6	8	4
Kyn	1.70 ± 0.35	2.42 ± 1.02*	2.83 ± 1.06	2.62 ± 1.11	2.59 ± 0.98	2.37 ± 1.15
Trp	52.55 ± 10.45	48.18 ± 16.56^*^	47.39 ± 13.74	49.11 ± 14.69	50.23 ± 15.72	51.37 ± 16.88
Kyn/Trp	0.032 ± 0.01	0.050 ± 0.028^*^	0.061 ± 0.029	0.053 ± 0.031	0.052 ± 0.033	0.046 ± 0.044

^*^Significant differences between TB patients and healthy controls, *P* < 0.05

## Discussion

IDO is associated with TB development [11, 12, 16], and Trp metabolites catalyzed by IDO activity can be used as biomarkers and prognostic factors in the diagnosis and prognosis of TB [13–15]. However, *IDO1* and *IDO2* expression and the effects of their SNPs on the host immune system in TB remain unclear. In this study, *IDO1* and *IDO2* SNPs in TB patients were compared with healthy controls, and only *IDO2* exhibited SNPs R248W(C–T) and Y359X(T–A) (Table 2). Different *IDO2* SNPs exhibited different serum Kyn/Trp ratios in TB patients, and participants with Y359X(AA) showed significantly lower IDO activity than participants with R248W(TT) (Table 4). This may be because SNP R248W reduced IDO2 catalytic activity and SNP Y359_stop_ generated a premature stop codon, completely abolishing catalytic activity [17]. Further research on *IDO2* SNPs related to specific diseases or symptoms can facilitate early diagnosis and immunotreatment of these diseases.

IDO-1-mediated tryptophan catabolism is highly conserved in the human response to *M. tuberculosis* [27]. However, IDO-1 deficiency fails to impact immune control and infection outcome in the mouse model of TB [12]. In addition, as in the *IDO2* genotypes, no *IDO1* SNP was observed in this study, irrespective of whether *IDO1* SNPs were associated with TB. However, *IDO1* SNPs have been linked to depression [20] and reduced susceptibility to recurrent vulvovaginal candidiasis [21]. IDO1 is expressed in various tissues and performs immunological functions in myeloid-derived suppressor cell development, pathogenic neovascularization, and immune tolerance [28]. IDO2 is crucial for autoantibody production and autoimmunity, and it activates B cells to regulate T-cell function [29]. Although *IDO2* is more narrowly expressed and less active than *IDO1*, *IDO2* and its SNPs may also play important inhibitory roles in autoimmunity.

IDO1 and IDO2 exhibit different enzyme activities [6, 7] and gene expression levels in various tissues [4, 5] and cell types [10, 28]. The qRT-PCR results confirmed that *IDO1* mRNA levels were higher in TB patients than in healthy controls, whereas *IDO2* mRNA levels followed the opposite trend (Fig. 3). In tumor tissues, IDO2 contributed to IDO1-mediated immune tolerance [4] and functioned as a negative regulator of IDO1 by competing with it for the heme binding site [9]. These findings indicate that IDO1 and IDO2 may interact and contribute to total IDO activity. The bioinformatics analysis suggested that IDO1 and IDO2 directly interacted to form a dimer (ΔG = − 12.75 kcal/mol; *K*_d_ = 4.50 e^− 10^ M). *IDO2* is uniquely regulated by the aryl hydrocarbon receptor, which serves as a physiological receptor for Kyn [28, 30]. In addition, the *IDO2* promoter includes a prominent binding site for the transcription factor interferon regulatory factor 7, a master regulator of dendritic cell maturation [31]. *IDO2* SNPs may affect IDO activity by binding to IDO1 through protein–protein interactions and competing with IDO1 for Trp.

The IDO–Kyn pathway is involved in TB by inducing strong expression of IDO1 and activation of Trp metabolism [12], but the role of IDO2 in TB is unclear. Higher IDO activity resulted in the active consumption of Trp in antigen-presenting cells and produced more Kyn, leading to a higher Kyn/Trp ratio [13, 14], which could be used as a biomarker in TB diagnosis [15, 16]. Moreover, Kyn activates FoxP3 expression and Treg cell differentiation in immune tolerance to pathogens [32], which further inhibited Th17 cell differentiation in the RORγt pathway. Because the number of Treg cells in peripheral blood mononuclear cells can be an indicator of tuberculin skin test reactivity and Bacillus Calmette-Guerin scar formation in TB patients [33–35], further clinical investigation is required to determine if the Treg/Th17 ratio may be a better index.

Limitations of this study were the small sample size of each SNP, the unknown severity of TB in patients (which may affect IDO activity), and no proof of direct interaction between IDO1 and IDO2. Therefore, the conclusions were reached by adopting a cautious approach.

## Conclusions

Only *IDO2* had SNPs R248W and Y359X that affected IDO activity. IDO activity is regulated by *Mycobacterium* infection and host gene polymorphisms, especially in *IDO1* and *IDO2*, and the balance of their expression in TB. This finding paves the way for future research on other pathologies that involve the IDO isoforms and sets the path for the discovery of inhibitors of not only IDO1 but also IDO2.

## Data Availability

All data and materials generated or analyzed during this study are included in the article.

## Abbreviations

TB: Tuberculosis
IDO: Indoleamine 2,3-dioxygenase
Trp: Tryptophan
Kyn: Kynurenine
SNPs: Single-nucleotide polymorphisms
